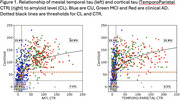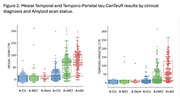# CenTauR correlation with amyloid, atrophy and cognition across the Alzheimer’s Disease spectrum: A tau imaging study with ^18^F‐MK6240 PET

**DOI:** 10.1002/alz70862_109695

**Published:** 2025-12-23

**Authors:** Christopher C. Rowe, Azadeh Feizpour, Pierrick Bourgeat, Antoine Leuzy, Rachel S Mulligan, Joanne Robertson, Simon M. Laws, Ralph N Martins, Paul Maruff, Colin L Masters, Jurgen Fripp, Victor L. Villemagne, Vincent Dore

**Affiliations:** ^1^ Department of Molecular Imaging & Therapy, Austin Health, Melbourne, VIC Australia; ^2^ Florey Department of Neuroscience and Mental Health, University of Melbourne, Parkville, VIC Australia; ^3^ The Florey Institute of Neuroscience and Mental Health, Parkville, VIC Australia; ^4^ CSIRO Health and Biosecurity, Australian E‐Health Research Centre, Brisbane, QLD Australia; ^5^ Lund University, Malmö Sweden; ^6^ Department of Psychiatry, Cognition and Aging Psychiatry, Sahlgrenska Academy, Region Västra Götaland Sweden; ^7^ Austin Health, Melbourne, VIC Australia; ^8^ The Florey Institute of Neuroscience and Mental Health, The University of Melbourne, Parkville, VIC Australia; ^9^ Collaborative Genomics and Translation Group, Edith Cowan University, Joondalup, Western Australia Australia; ^10^ School of Medical and Health Sciences, Edith Cowan University, Perth, Western Australia Australia; ^11^ Centre for Ageing, Cognition and Wellbeing, Macquarie University, North Ryde, NSW Australia; ^12^ Cogstate Ltd., Melbourne, VIC Australia; ^13^ The Florey Institute of Neuroscience and Mental Health, The University of Melbourne, Parkville, Melbourne, VIC Australia; ^14^ University of Pittsburgh School of Medicine, Pittsburgh, PA USA; ^15^ The Australian e‐Health Research Centre, Commonwealth Scientific and Industrial Research Organisation, Brisbane, QLD Australia; ^16^ Department of Molecular Imaging, Austin Health, Melbourne, VIC Australia

## Abstract

**Background:**

The CenTauR (CTR) standard method for PET measurement of tau provides consistent results across tau tracers. This study establishes regional CTR thresholds for elevated tau and evaluates the CTR relationship with amyloid, neurodegeneration and cognition across the AD spectrum.

**Method:**

924 participants from the AIBL‐ADNeT study (448 cognitively unimpaired (CU), 300 MCI, 176 clinical AD), underwent ^18^F‐MK6240 tau PET, ^18^F‐NAV4694 Aβ PET, MRI and cognitive evaluations. We examined the relationship between global and regional tau quantification using the CTR method, and global Aβ (Centiloid), brain atrophy and cognition.

**Result:**

From tau scans in A‐ (<25 CL) CU, 2 SD thresholds were 11 CTR in the mesial temporal lobe (MTL) and 15 CTR in the Temporo‐Parietal (TP) cortex. There was a non‐linear relationship between Aβ and tau with cortical tau rare below 60 CL (Figure 1). In clinical AD, 71% had A+ and neocortical tau+ (T_TP_+), 14% had neither, 14% had only A+. In MCI, 43% had both, 30% had neither and 26% had only A+. In CU, 64% had neither, 27% were A+T‐, and 7% A+T_TP_+. However 31% of CU, 68% of MCI and 84% of clinical AD were T_MTL_+. A‐T_MTL_+ was observed in 9% of CU, 8% of MCI and 3% of clinical AD (Figure 2). In A+ MCI and AD, cortical tau was strongly inversely associated with age and cortical volume. No association was found with hippocampal volume when tau was limited to the MTL. The most significant factors associated with worse cognition were age and male sex in CU; Aβ, hippocampal volume and tau in MCI, and tau and hippocampal volume in clinical AD. Cortical tau correlated with worse general cognition, while tau limited to the MTL only affected episodic memory. In the absence of tau, Aβ did not correlate with cognitive decline.

**Conclusion:**

This study has established regional CTR thresholds and the prevalence of MTL and cortical tau in CU, MCI and clinical AD. It has confirmed that cortical tau is rarely found below 60 CL of Aβ. Cortical CTR strongly correlates with cognitive impairment and atrophy, while tau restricted to the MTL is linked to mild memory impairment.